# Distract yourself: prediction of salient distractors by own actions and external cues

**DOI:** 10.1007/s00426-018-1129-x

**Published:** 2018-12-26

**Authors:** Ondřej Havlíček, Hermann J. Müller, Agnieszka Wykowska

**Affiliations:** 1 Department of Psychology, General and Experimental Psychology Unit, Ludwig-Maximilians-University of Munich, Leopoldstr. 13, 80802 Munich, Germany; 2 Max Planck Institute for Human Cognitive and Brain Sciences, Stephanstraße 1a, 04303 Leipzig, Germany; 3 Graduate School of Systemic Neurosciences, Ludwig-Maximilians-University of Munich, Großhaderner Str. 2, 82152 Planegg-Martinsried, Germany; 4 Department of Psychological Sciences, Birkbeck College, University of London, Malet Street, WC1E 7HX London, UK; 5 Research line “Social Cognition in Human-Robot Interaction”, Istituto Italiano di Tecnologia, Via Morego, 30, 16163 Genova, Italy

## Abstract

Distracting sensory events can capture attention, interfering with the performance of the task at hand. We asked: is our attention captured by such events if we cause them ourselves? To examine this, we employed a visual search task with an additional salient singleton distractor, where the distractor was predictable either by the participant’s own (motor) action or by an endogenous cue; accordingly, the task was designed to isolate the influence of motor and non-motor predictive processes. We found both types of prediction, cue- and action-based, to attenuate the interference of the distractor—which is at odds with the “attentional white bear” hypothesis, which states that prediction of distracting stimuli mandatorily directs attention towards them. Further, there was no difference between the two types of prediction. We suggest this pattern of results may be better explained by theories postulating general predictive mechanisms, such as the framework of predictive processing, as compared to accounts proposing a special role of action–effect prediction, such as theories based on optimal motor control. However, rather than permitting a definitive decision between competing theories, our study highlights a number of open questions, to be answered by these theories, with regard to how exogenous attention is influenced by predictions deriving from the environment versus our own actions.

## Introduction

Perceptual processing to perform a task often faces interference from various kinds of distracting stimuli. A classic example is that when we are searching for some visual target, a task-irrelevant but salient distractor interferes with (e.g., slows) search performance—a phenomenon referred to as involuntary ‘attentional capture’ (e.g., Hickey, McDonald, & Theeuwes, 2006; Theeuwes, 1992; Theeuwes, Atchley, & Kramer, 2000; Yantis, 1993). There has been considerable debate as to whether and how such capture events may be reduced (Bacon & Egeth, 1994; Christ & Abrams, 2006; Eimer & Kiss, 2008; Müller, Geyer, Zehetleitner, & Krummenacher, 2009; Müller, Reimann, & Krummenacher, 2003; Theeuwes, 2010; Wykowska & Schubö, 2010, 2011). One possible way for top-down processes to modulate bottom-up capture may be based on predictive information regarding aspects of the distracting stimuli.

## Attentional white bear? Predicting the irrelevant item

Conceivably, knowing about the location or defining feature(s) of a distracting item might help ignore it; though, paradoxically, attention might also be especially drawn to this item, increasing its distracting effect. The latter effect has actually been reported in the literature (Huffman, Rajsic, & Pratt, 2017; Lahav, Makovski, & Tsal, 2012; Tsal & Makovski, 2006) and termed ‘attentional white-bear’ phenomenon (AWB; Tsal and colleagues) or ‘ironic capture’ (Huffman and colleagues). Tsal and colleagues argued that the first item selected is likely to be the distractor, in part because the very instruction to ignore the distractor will represent it, as a kind of ‘template’, in visual working memory (vWM), biasing the allocation of attention towards a distractor appearing in the display—in the same way as trying not to think about a white bear makes one focus on its very mental image.

Recent explorations of the AWB or ‘ironic-capture’ hypothesis have yielded conflicting findings. Several studies reported that providing various kinds of information about the distractor (e.g., its defining feature to be held in vWM) increased attentional capture (Beck, Luck, & Hollingworth, 2011; Olivers, 2009), whereas other studies found participants to be able to use distractor features to reduce capture (Arita, Carlisle, & Woodman, 2012; Dhawan, Deubel, & Jonikaitis, 2013; Woodman & Luck, 2007). In addition, while even task-irrelevant symbolic spatial cues can give rise to attentional orienting (Hommel, Pratt, Colzato, & Godijn, 2001), predicting the location of a distractor by means of presenting participants with an advance spatial cue, or as a result of statistical learning, has been reported to be beneficial to performance (Chao, 2010; Munneke, Van der Stigchel, & Theeuwes, 2008; Ruff & Driver, 2006; Sauter, Liesefeld, Zehetleitner, & Müller, 2018). Others, by contrast, have failed to find suppression of distractor locations in response to spatial cues (Buckolz, Guy, Khan, & Lawrence, 2006).

## Prediction by action

In almost all studies on this issue thus far, predictive information about the distractor was ‘external’ in nature, for instance, in the form of an explicit spatial cue indicating the distractor location. A neglected, though at least as important source of predictions about upcoming sensory events, are our own actions (Waszak, Cardoso-Leite, & Hughes, 2012): throughout our lives, we learn which sensory outcomes result from motor actions we perform. Compared to external cues, actions are generally thought to involve specific predictive information: information that enables us to distinguish self- from environment-produced effects, thus contributing to a sense of agency and a rudimentary sense of self (Gallagher, 2000). Accordingly, it would be reasonable to expect that predictability of the sensory consequences of actions can be utilized to better guide attention to task-relevant target and away from distracting stimuli (e.g., when honking the horn of a car, we do not get distracted by the horn’s sound because we caused it ourselves). However, little is known as yet about the specific impact of action–effect prediction on visuo-spatial attention and the mechanisms involved.

It is generally thought that action-based predictions attenuate the strength of the actions’ sensory consequences: a phenomenon that has been called ‘sensory attenuation’ (Waszak et al., 2012; Wolpert & Flanagan, 2001). A paradigmatic case in point is that we find it hard to experience the sensation of being tickled when we ourselves control a robotic arm that does the tickling, whereas we have more of a feeling of being tickled if a temporal delay or trajectory perturbation is introduced into the motion of the robotic arm (Blakemore, Frith, & Wolpert, 1999). While sensory attenuation has also been demonstrated in the auditory domain (Baess, Horváth, Jacobsen, & Schröger, 2011; Hughes, Desantis, & Waszak, 2013; Weiss, Herwig, & Schütz-Bosbach, 2011), there is a paucity of literature as regards the visual domain. A study by Cardoso-Leite and colleagues reported a decrease in sensitivity for self-produced visual stimuli (action-related prediction), compared to stimuli predicted by auditory tones (accompanied by a non-predictive action) (Cardoso-Leite, Mamassian, Schütz-Bosbach, & Waszak, 2010).

Traditionally, explanations of sensory attenuation are based on the optimal motor control theory (Blakemore et al., 1999; Wolpert & Flanagan, 2001). These explanations posit that when we act, an efference copy of the motor command enters a forward model, which predicts the sensory consequences of the action (e.g., a salient distractor) and this prediction (corollary discharge) is then compared with (and subtracted from) the actual sensory consequence, attenuating its strength. A distractor predicted by an action should thus produce less interference compared to a non-predicted distractor or a distractor predicted solely by external events, or cues, which would not enter the forward model in the same way. There is another group of theories relating to the notion of predictive processing which attempts to explain sensory attenuation in a more general way (Brown, Adams, Parees, Edwards, & Friston, 2013; Clark, 2013; Friston, 2011; Pickering & Clark, 2014; Van Doorn, Hohwy, & Symmons, 2014), which will be discussed later, see the “Discussion” section.

## Aim of study

On this background, the present study was designed to examine two related questions: First, would predictability of a highly salient but task-irrelevant visual stimulus through participants’ own actions increase or decrease the interference it generates under conditions of ‘efficient’ visual search, that is, when both the target and the distractor ‘pop out’ and, thus, strongly compete for selection? Second, can the effects of prediction be attributed to motor-related prediction processes (rather than, e.g., a more general prediction process)?

## Design

To investigate these questions, we adopted a commonly used paradigm for investigating the influence of salient distractors: visual search for a salient singleton target with an additional, irrelevant salient singleton (i.e., the distractor) in the display. The task required participants to respond to a non-defining feature of the singleton target (the so-called ‘compound’ search task, cf. Duncan, 1985). Using this paradigm, we manipulated the (joint) predictability of distractor presence and location in three conditions: baseline (no prediction), cue prediction, and action prediction.

As pointed out by Hughes et al. (2013), many studies investigating the influence of action-driven prediction on perception might actually be confounded, because compared to the usual control conditions, the participants’ action did not just predict the identity of the resulting stimulus (specific configuration and properties of items) but it also allowed for temporal prediction as to when the stimulus would appear, that is, temporal control over the (onset of the) stimulus. Furthermore, the mere presence of an action might influence cognitive processes other than those related to action–effect prediction. Finally, people may use predictive strategies that do not rely on the motor information, such as simply using the knowledge that certain effects usually follow certain actions. Given all this, we devised our conditions such as to carefully control for these confounds (Table 1). Compared to the baseline, our cue prediction condition was designed to isolate only the specific influence of the predictive information conveyed by the cue—that is, “non-motor identity prediction” processes in the terminology of Hughes et al. (2013). And our action prediction condition, when compared to the cue prediction condition, was designed to isolate only the specific influence of what Hughes et al. (2013) refer to as “motor identity prediction” processes, such as the forward model processes, which were the main focus of our study. To control for “temporal prediction” and “temporal control” as well as the mere presence of action, the same actions were used to trigger the stimuli in all three conditions; however, only in the action prediction condition were the actions predictive as to the presence and location of a distractor.

**Table 1:** Contrasts between the prediction processes involved in the baseline, action and cue prediction conditions; based on Hughes et al. (2013)

Condition or contrast type	Temporal prediction	Temporal control	Non-motor identity prediction	Motor identity prediction
(B)aseline	*	*		
(C)ue	*	*	*	
(A)ction	*	*	*	*
Contrast (C)–(B)			*	
Contrast (A)–(C)				*

In the action prediction condition, the sensorimotor contingencies between action and stimulus were arbitrary. This meant they had to be learned by the participants in an ‘association phase’ that directly preceded the action prediction condition proper (Herwig & Waszak, 2012; Richters & Eskew, 2009), through repeated coupling of an action (a ‘cause’: a button press with the left or the right hand) with a stimulus (an ‘effect’, distractor presented at one of two spatial locations). This necessitated constraints on the order in which the three prediction conditions could be administered, specifically: the other two (i.e., baseline and cue prediction conditions) could not be performed after participants had acquired this association (after the action prediction condition), because—for the reasons outlined above—the very same actions (button presses with the left/right hand, though not coupled with particular distractor effects) were used in those conditions as well. Given this, performing these conditions after the action–effect learning would have confounded the results. Therefore, we presented the three conditions in a fixed, sequential manner (see below). Note that encountering distractors in mere practice trials before performing the visual search experiment proper already helps participants reduce the interference caused by the distractors (Müller et al., 2009). Given this, since prior exposure to the distractor stimuli was an inherent part of the association phase in the action prediction condition (distractors had to be shown to be associated with the actions participants performed), this factor also needed to be controlled for in the other two conditions—namely, by simply introducing distractor exposure phases prior to the baseline and the cue prediction conditions. The order of phases and conditions was thus as follows: exposure phase → baseline condition → exposure phase → cue prediction condition → association phase → action prediction condition.

**Fig. 1:**
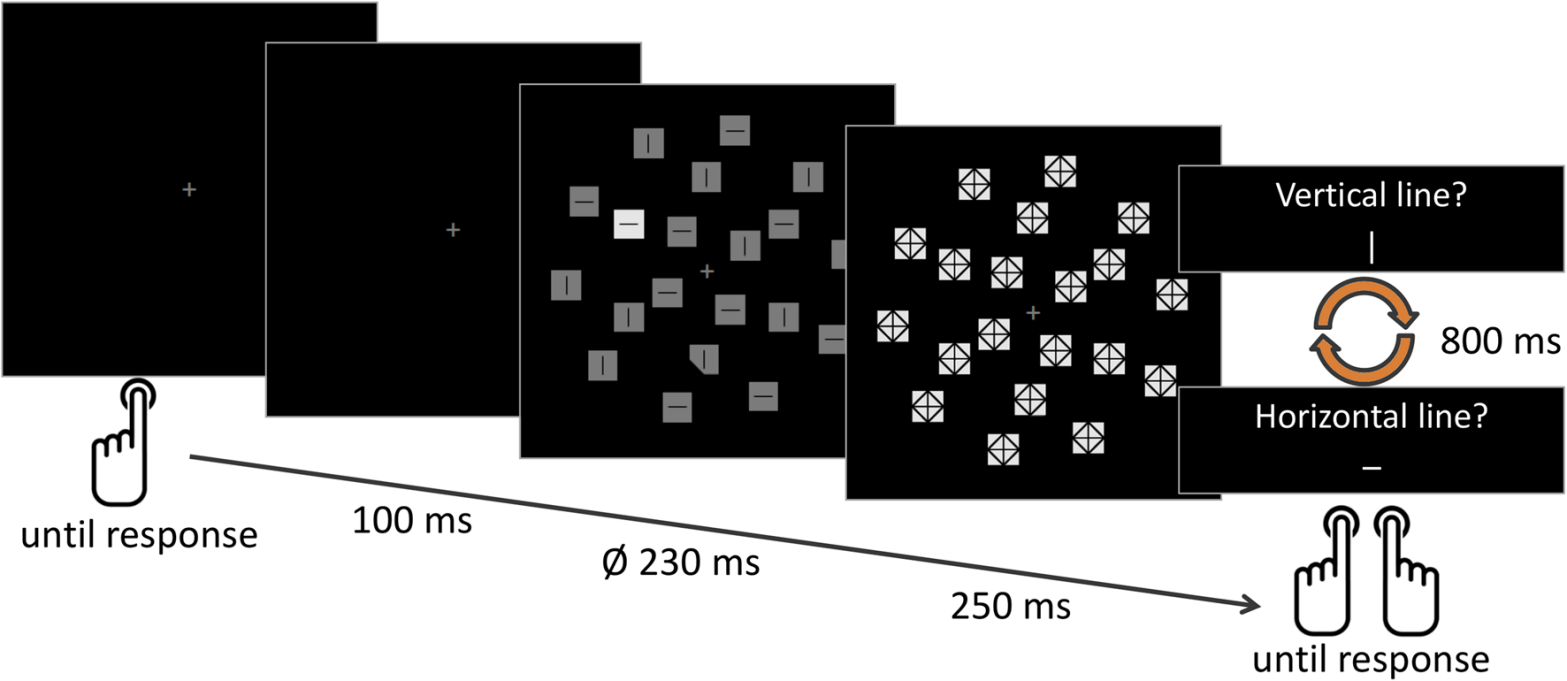
Basic trial sequence. Each trial began with a fixation cross (or a cue, in the cue prediction) displayed until the participant pressed the left, right, or neutral key. This triggered the presentation of the search display, which was shown for a duration previously determined by a staircase procedure and then masked. Next, participants responded by selecting the target probe orientation (i.e., the orientation of the line inside the cut-off grey square) from two alternating response options, by pressing the neutral key with both hands

To avoid interference of the response required by the task (compound task requiring a two-alternative choice response to the critical target property) with the learned action–effect associations, we adopted the same procedure as Cardoso-Leite et al. (2010) in all conditions: following the search display, two alternative response options were presented alternately on the screen (one at a time) until the participant stopped the alternation by issuing a neutral action (using both hands at the same time to press a button, thereby selecting one of the response options; see also Fig. 1). Given that this response procedure does not allow for speeded reactions required for measuring reaction times (RTs), only performance accuracy was available as dependent variable in Experiment 1.

Of note, distractor interference has been hitherto most reliably observed in terms of slowed RTs (Theeuwes, 1992; Yantis, 1993). Nevertheless, assuming that the RT cost generated by the distractor originates from the process of visual selection (rather than from, e.g., response selection), presenting the search display only briefly (threshold duration determined by a staircase procedure) and terminating its exposure by presenting post-display masks would make it less likely for the target to be processed if attention had first been captured by the distractor on a fraction of trials (Zehetleitner, Koch, Goschy, & Müller, 2013). Consistent with this, Kiss, Grubert, Petersen, and Eimer (2012) reported increased error rates owing to distractor presence in displays presented for 200 ms (though no masks were used in their paradigm). On the other hand, using a similar paradigm but with search displays presented for only 86 ms and then masked, Gibson and Jiang (1998) failed to find a significant cost in accuracy—their only dependent measure. However, this is likely attributable to the fact that their search task was very ‘inefficient’, requiring serial scanning of display items.

Given that demonstrating distractor interference with short presentation times has proved difficult in the past, we created conditions of strong overall distractor interference—thus making it more likely for interference to be reflected in performance accuracy. Specifically, in the compound search task employed, the target-defining feature was an odd-one-out shape, while the response was to be made with respect to the orientation of a line probe inside the target shape. Defining the target as a singleton shape was expected to increase participants’ reliance on a ‘singleton search’ strategy, under which the interference from singleton distractors is assumed to be maximal (Bacon & Egeth, 1994; Lamy & Egeth, 2003). The irrelevant singleton was made more salient than the target by virtue of its increased luminance—based on the assumption that sensory attenuation can more easily influence the perceived intensity of stimulus compared to, for instance, its perceived shape or color (luminance varies along a single ‘dimension’, so it may be easier to ‘subtract’ some luminance; shape or color, by contrast, are complex dimensions, so that one cannot ‘subtract’ in the same way). In addition, we used dense displays with both target and distractor completely surrounded by neutral, ‘non-target’ items to further increase the salience of the singleton distractor (Rangelov, Müller, & Zehetleitner, 2013, 2017). The distractor could appear at only one of two possible locations, so that we could associate these two locations with two different actions.

## Experiment 1

### Methods

#### Participants

Because the task was rather difficult, data collection was ongoing until we had usable datasets from 30 participants. The criterion for this was defined a priori as accuracy above chance level in each combination of predictive condition and distractor presence. Overall, 44 participants were tested, but 14 failed to meet this criterion. Participants were randomly assigned to one of two action–effect contingency groups (described below), with 15 participants in each group. The number of participants was based on the expected effect size that action prediction should have on top of cue prediction according to theories invoking action–effect prediction via forward models. To our knowledge, there is only one study comparable in its aim and design (Cardoso-Leite et al., 2010), based on which we estimated the effect size as *d*_z_ = 0.546^1^. To detect an effect of such size with a reasonable power of at least 0.80 in a within-participants design, we would need to test 29 participants. Because we use a subdivision into two groups of participants, we decided for a sample size of 30 participants.

Participants’ age range was 19–34 (*M* = 24.6) years; all of them were right-handed, and nine were male. All participants reported normal or corrected-to-normal vision. They were paid € 8 per hour or opted to receive a course credit. The experiments were conducted at the Experimental Psychology Laboratory of the LMU Munich. All experimental procedures consisted of purely behavioral data collection with healthy adult participants and did not involve any invasive or potentially dangerous methods. The study was approved by the Ethics Committee of the LMU Psychology Department, in accordance with the Code of Ethics of the World Medical Association (Declaration of Helsinki). Data were stored and analyzed anonymously. All participants provided written, informed consent.

#### Apparatus and stimuli

Participants were seated in a dimly lit and sound-attenuated room, in front of a CRT monitor (LaCie Electron 21/108, screen refresh rate 100 Hz, screen resolution 1024 × 768 pixels) at a viewing distance of 58 cm (maintained using a chin rest). A standard keyboard was used to collect responses. Participants were instructed to use their left middle finger to press the C key (left response key), the right middle finger to press M key (right response key), and to press the spacebar always using both index fingers at the same time for a neutral response.

The E-Prime software (Psychology Software Tools Inc., Sharpsburg, PA, version 2.0 Professional) was used to set up and present the stimuli. The search display consisted of 20 gray square items (size 1.05° × 1.05° of visual angle, luminance 13.3 cd/m^2^, RGB [64, 64, 64]) against a black background (luminance 1.24 cd/m^2^); the items were positioned around three (imaginary) concentric circles (equally spaced, outer diameter 11.7°) with a gray fixation cross in the center. In the search displays, one of the items (the target) had one of the four corners cut off. On some trials, a bright gray square (a distractor) was present [luminance 58.4 cd/m^2^, RGB (160, 160, 164)]. Additionally, each of the items contained a probe: a black line (size 0.6 × 0.1°) oriented either vertically or horizontally. The target and the distractor were limited to locations on the middle circle (diameter 7.2°). Pattern masks were presented at the end of display exposure. The masks consisted of a black-line cross and a diamond inside a square (to mask probe lines as well as the contour line produced by cutting off one corner of the target stimulus) and was of the same color and luminance as the distractor (see Fig. 1 for a depiction).

#### General procedure

The experiment consisted, essentially, of the three blocked conditions: baseline, cue prediction, and action prediction, in which participants performed a variation of essentially the same task; this task will be described first, followed by the specific differences among the three conditions and other details.

#### Visual search task

Each trial began with a gray fixation cross in the center of the screen. Participants could then, at any time, press a key—which, after a delay of 100 ms, produced the search display (Fig. 1). Each item in the display contained a line probe oriented randomly in either vertical or horizontal direction. Participants’ task was to search for a shape singleton (target)—a square with a random corner cut off—and report the orientation of the line inside this shape. The target could appear at one of the six locations on the middle circle, twice as likely at the top and bottom locations, relative to the lateral positions. (This specific ratio was chosen to allow for a comparison with a planned ERP study, which would require such a ratio of midline and lateral target occurrences.) In one-half of the trials, a luminance distractor was randomly displayed at either the top-left or bottom-right location. Participants were instructed to ignore the distractor. The search display was presented only for a brief period of time, determined individually by a pre-experimental staircase procedure (*M* = 227 ms, SD = 83). The display was then masked for 250 ms. Next, the response options “horizontal line?” and “vertical line?”, with a picture of the respective line orientation, were presented alternately on the screen (800 ms per option) until the participant selected one option by pressing the neutral key using both hands (the selected option was the one displayed when the neutral key was pressed). Feedback was provided in the case of an incorrect response (in the form of a red “minus” sign presented for 1000 ms). Afterwards, a blank screen was displayed for an inter-trial interval (ITI) of 250–550 ms (uniform random distribution).

Participants were asked to press one of three different keys to initiate each trial: the left, the right, or the neutral key. They were instructed to choose among the keys at will, but to press the neutral key about twice as often as the other keys, optimally in a ratio of 25%:25%:50%. In the baseline and cue prediction conditions, which key was selected had no implication on the task. Making participants perform the initial key-press action in the above ratio served two purposes: learning this ratio for the action prediction condition and equating the cognitive demands and level of alertness and preparedness for the upcoming trial among all three conditions. In the cue prediction condition, the fixation cross at the start of a trial was replaced by a central symbolic cue, either a left arrow (<) sign, indicating that the distractor would be displayed at the top-left location; or a right arrow (>) sign, indicating a distractor at the bottom-right location; or a minus (–) sign, indicating that no distractor would be presented. The cue was displayed until a participant initiated a trial with a button press, and it was 100% valid. Participants were explicitly informed about this and told to use the information provided by the cue in any way that could help them perform the task better. In the action prediction condition, the key used to start the trial determined the presence and location of the distractor. The neutral key produced no distractor, while the left and right keys would produce a distractor at one of the two usual (i.e., the top-left or bottom-right) locations. This action–effect contingency was counterbalanced across participants (between-participants factor “contingency group”: natural mapping versus inverse mapping): for one-half of the participants, the left key would produce the distractor at the top-left position and the right key the distractor at the bottom-right position, and vice versa for the other half. Participants were also explicitly informed about this.

There were six blocks of trials in each of the three conditions, each block consisting of 32 trials, yielding a total of 192 trials per condition. After each block, participants were given a feedback about their key press ratio and allowed to rest for a while.

#### Association task

The action prediction condition was preceded by an association phase, to permit participants to learn the sensorimotor contingencies between an action (button press) and the observed effect (display with a distractor) prior to performing the action prediction condition proper. The task in the association phase was to randomly press the left or the right key—in a ratio of approximately 50%:50%, at a pace of about one press every two seconds—while an empty screen with just a fixation cross was displayed. The key press produced (after a delay of 100 ms) a display that was similar to the search display in the search task proper, except that it always contained a distractor singleton, but no target, and there were no probe lines inside the items. The distractor appeared at one of the two possible locations, according to the participant’s contingency group. The duration of this display was 600 ms. To ensure that participants payed attention to these displays, the central fixation cross was red in one-eighth of the trials. On such catch trials, participants were required to immediately press the neutral key with both their index fingers at the same time. The response window for the catch trials was 1000 ms. In case of an incorrect response or a failure to respond, a red “minus” sign would appear for 1000 ms.

There were seven blocks of trials, each block consisting of 64 trials, that is, 448 association trials in total. The number of association trials was chosen based on the Cardoso-Leite et al. (2010) study. After each block, participants were given feedback about their key press ratio and allowed to rest.

#### Exposure task

An exposure phase was administered before both the baseline and the cue prediction conditions. This phase was the same as the association phase, but instead of participants starting the trials with a button press, the displays appeared on their own after 600 ms. There were six blocks of exposure trials, each block consisting of 64 trials, that is, 384 exposure trials in total.

#### Staircase

Before the actual experiment, the search display durations were determined individually for each participant. An adaptive staircase procedure was used to find the individual thresholds. The visual search task described above was used; however, only the neutral key was used to start the trials and a distractor was always present, located randomly at any of the six locations on the middle circle. The search display duration started at a set value of 400 ms and was increased by one step size in case of an error and decreased by one step size in case of two successive correct responses. This staircase rule aimed at an accuracy threshold of approximately 71%. Step size was 80 ms until the 4th reversal point (error after a correct response or vice versa), 40 ms until the 6th reversal, and then kept at 10 ms. The procedure terminated after 16 reversals, and the final display duration was calculated as the average duration across the last 10 reversal points, rounded to a multiple of ten.

#### Overall structure of the experiment

Participants began with the staircase phase to establish the display duration to be used in a subsequent practice phase. This practice phase had the same structure as the actual experiment but was limited to two blocks of eight trials per each of the six experimental phases. After practice, participants performed the staircase procedure once more, and the value obtained was introduced in the actual experiment. After the experiment, a one-question “questionnaire” was administered asking participants: In what way did you use the information provided by the cue? The whole experiment took between 1.5 and 2 h to complete, including instructions and all breaks.

#### Analysis

To verify that participants were actually able to perform the main task above chance level, individual performance was assessed using a binomial test for each combination of prediction-type condition and distractor presence. If the accuracy in any of these combinations was not significantly higher than expected by chance (*α* = 0.05), the data of this participant were excluded from analysis. Additionally, several trials in the action prediction phase had to be excluded due to technical issues (error in the program) during data acquisition. However, this affected only 2.57% of the trials, on average, in this particular condition.

We tested our hypotheses using a 2 × 3 repeated-measures analysis of variance (ANOVA) on mean accuracies, with the factors ‘prediction type condition’ (baseline, cue prediction, action prediction) and ‘distractor presence’ (distractor absent, distractor present), followed up with individual two-tailed paired-samples *t* tests comparing the cost of distractor presence on accuracy between prediction type conditions. Of most interest to our first main question—whether the distractor would exert a lesser or greater influence on search performance when predicted—was the difference in distractor interference between the baseline and each of the two prediction-type conditions. Our second question—that is, whether the effect of prediction can be attributed to motor-related prediction processes—was examined by analyzing the difference in distractor interference between the two prediction-type conditions. Distractor interference was quantified as the difference in accuracy between distractor-absent and distractor-present trials.

### Results

**Fig. 2:**
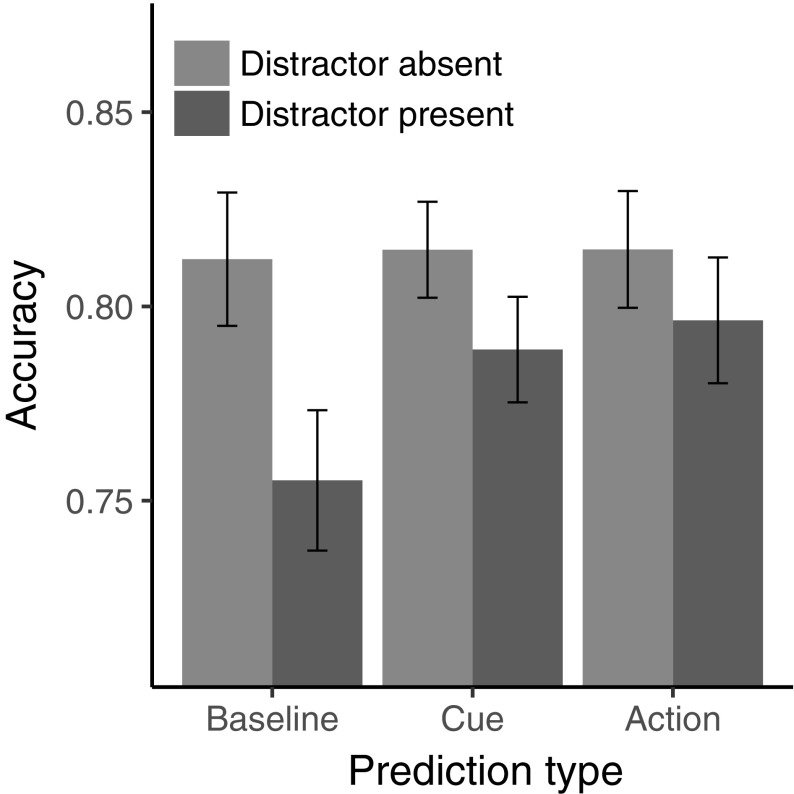
Main results of Experiment 1. Performance accuracy (proportion of correct responses) per prediction type and distractor presence conditions. Error bars depict 95% confidence intervals for the mean corrected for dependence in measurements (Morey, 2008)

The ANOVA (described above) revealed a significant main effect of distractor presence, *F*(1, 29) = 21.0, *p* < 0.001, $\eta _{{\text{G}}}^{2}$ = 0.048, $\eta _{{\text{p}}}^{2}$ = 0.420: participants exhibited generally lower accuracy in the presence of a distractor (*M* = 0.780, SD = 0.075) compared to its absence (*M* = 0.814, SD = 0.077). Follow-up *t* tests revealed that the distractor interference was significant not only in the baseline condition (95% CI for the difference between means = [0.031, 0.083], *t*[29] = 4.449, *p* < 0.001, *d*_z_ = 0.812) but also in the cue prediction condition (95% CI = [0.0065, 0.045], *t*[29] = 2.743, *p* = 0.010, *d*_z_ = 0.501), while being marginal in the action prediction condition (95% CI = [− 0.0019, 0.038], *t*[29] = 1.85, *p* = 0.074, *d*_z_ = 0.339); see Table 2. Furthermore, there was a significant main effect of prediction type, *F*(2, 58) = 3.676, *p* = 0.031, $\eta _{{\text{G}}}^{2}$ = 0.016, $\eta _{{\text{p}}}^{2}$ = 0.112, see Table 2. Importantly for the purpose of the present study, the interaction between distractor presence and prediction type was significant, *F*(2, 58) = 4.509, *p* = 0.015, $\eta _{{\text{G}}}^{2}$ = 0.012, $\eta _{{\text{p}}}^{2}$ = 0.135 (see Fig. 2), that is, predictability of the distractor did modulate its detrimental influence.

**Table 2:** Performance accuracy (means and standard deviations) for all prediction-type and distractor present conditions

Prediction-type condition	Overall		Distractor absent		Distractor present		Interference size	
	M	SD	M	SD	M	SD	M	SD
Baseline	0.784	0.078	0.812	0.074	0.755	0.071	0.057	0.070
Cue	0.802	0.071	0.815	0.070	0.789	0.071	0.026	0.051
Action	0.806	0.084	0.815	0.088	0.796	0.079	0.018	0.054

*M* mean, *SD* standard deviation, *N* = 30, values represent accuracy, i.e., the proportion of correct responses

To directly address whether the type of prediction had an influence on the reduction of attentional capture, the interaction was followed up with *t* tests on the size of distractor interference (mean accuracy on distractor-present trials minus mean accuracy on distractor-absent trials). There was a significant difference in the magnitude of distractor interference (1) between the baseline and the cue prediction condition (95% CI = [0.00032, 0.062], *t*[29] = 2.07, *p* = 0.048, *d*_z_ = 0.377), with interference being less marked in the latter condition; and (2) between the baseline and the action prediction condition (95% CI = [0.0095, 0.068], *t*[29] = 2.71, *p* = 0.011, *d*_z_ = 0.495), again with less marked interference in the latter condition—see Table 2. This pattern indicates that both kinds of predictive information were effective in attenuating distractor interference. However, the difference in distractor’s interference between the cue and action prediction conditions was not significant, 95% CI = [− 0.016, 0.031], *t*[29] = 0.656, *p* = 0.517, *d*_z_ = 0.120. Note that the predictive information influenced mainly the distractor-present trials; there was no significant difference among the prediction-type conditions for distractor-absent trials (*F*[2, 58] = 0.037, *p* = 0.963, $\eta _{{\text{G}}}^{2}$ < 0.001, $\eta _{{\text{p}}}^{2}$ = 0.001).

For additional analyses (learning effects, effects of sensorimotor contingencies, and item location and distance effects), see the Appendix.

One of our main questions concerns the difference in distractor interference between prediction based on a cue versus an action. Since we did not observe a statistically significant difference between these two conditions (*p* = 0.517), we cannot make any firm conclusions as to the actual presence or absence of the effect. However, we can analyze the likelihood of having obtained a false negative finding, given that we had an a priori expectation for the effect size of *d*_z_ = 0.546 (Cardoso-Leite et al., 2010). First, our achieved statistical power for an effect of such a size is 0.824, which makes the chances of a false negative finding relatively small, without, however, eliminating such a possibility. Second, a Bayes factor analysis using a Cauchy prior on standardized effect size with a recommended scale *r* = 0.707 to allow for a wider range of expected effect sizes (Rouder, Speckman, Sun, Morey, & Iverson, 2009) indicated that there is 4.22 times more evidence for the null hypothesis of no effect.

However, it may simply be the case that the effect is smaller than expected or that accuracy was not a sufficiently sensitive measure. For this reason, we conducted a follow-up experiment which used reaction times as the main dependent measure. This follow-up experiment was limited to exploring the currently observed null difference between the cue and action prediction conditions, and thereby included only the two conditions of interest.

## Experiment 2

### Methods

#### Participants

Experiment 2 was conducted at the Istituto Italiano di Tecnologia, Genova, Italy. Twenty-eight new participants took part, receiving an honorarium for their service. Participants were randomly assigned to one of two action–effect contingency groups as in the first experiment. Furthermore, in each group half of participants started with the cue prediction condition and the other half with action prediction condition. One participant was excluded due to chance-level performance. Participants’ age range was 18–31 (*M* = 25.7) years; two were left-handed, and 13 were male. All participants self-reported normal or corrected to normal vision.

Written informed consent was given by each participant. The study was approved by the local ethical committee (Comitato Etico Regione Liguria) and was conducted in accordance with the Code of Ethics of the World Medical Association (Declaration of Helsinki). Data were stored and analyzed anonymously.

#### Procedure

The procedure was generally the same as in Experiment 1, though measuring reaction times necessitated a few modifications. In particular, we focused solely on the cue and action prediction conditions, presenting them in counterbalanced order across participants. For this reason, participants now also started the exposure trials preceding the cue prediction phase by randomly pressing the left or right keys, as in the action–effect association phase (that preceded the action prediction phase), but the subsequently displayed distractor appeared randomly on the left or the right side, in order for participants to unlearn any action–effect associations they may have had acquired previously. The second modification related to task response, which became speeded. Hence, there was no staircase procedure; search displays were presented until response, and there were no post-display masks; participants had to use two different response options to indicate the target probe line orientation as fast and as accurately as possible. The search display was again started by a keyboard press and the search display appeared after 100 ms. Participants issued their target orientation responses by pressing one of two foot pedals. Responses were given via foot pedals because we deemed it potentially confusing for participants (and giving rise to interference) had they had to produce another keyboard response so shortly after initiating the trial by a manual key press.

### Results

**Fig. 3:**
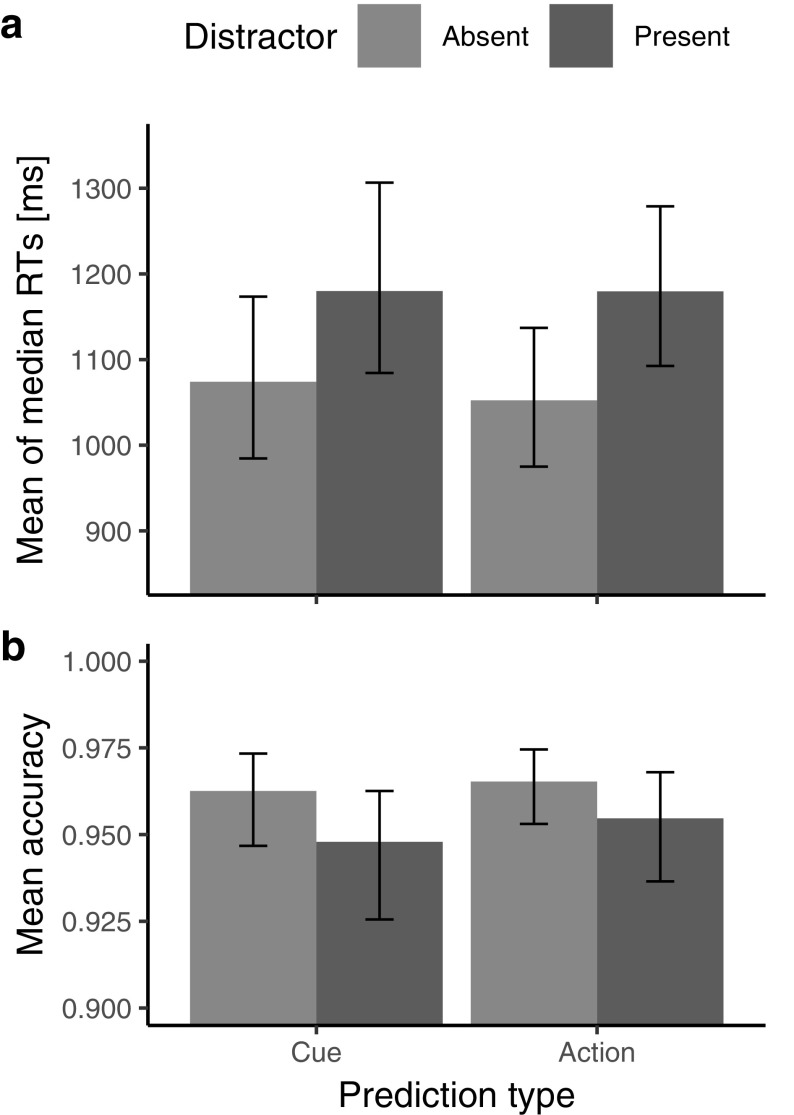
Results of experiment 2. Mean of individual medians of reaction times (**a**) and mean of individual proportions of correct responses (**b**) for each prediction-type and distractor presence. Error bars depict bootstrapped 95% confidence intervals for the mean

Accuracy was generally at ceiling level; 95% CI for the mean of individual accuracies = [0.943, 0.968]. An ANOVA on the individual accuracies yielded a significant main effect of distractor presence (*F*[1, 26] = 13.5, *p* = 0.0011, $\eta _{{\text{G}}}^{2}$ = 0.026): slightly more errors were made when a distractor was absent rather than present (0.964 vs. 0.951); critically, however, neither the main effect of prediction type nor the prediction type × distractor presence interaction was significant (both F[1, 26] < 0.8, p > .39). Similarly, an ANOVA on the medians of the individual reaction times (RTs) revealed a significant main effect of distractor presence (F[1, 26] = 63.8, *p* < 0.001, $\eta _{{\text{G}}}^{2}$ = 0.050): RTs were overall slower on distractor-present (*M* = 1168, SD = 244 ms) compared to distractor-absent trials (*M* = 1061, SD = 237 ms). However, neither the main effect of prediction type (*F*[1, 26] = 0.11, *p* = 0.743, $\eta _{{\text{G}}}^{2}$ = 0.0005) nor the prediction type x distractor presence interaction (*F*[1, 26] = 1.06, *p* = 0.312, $\eta _{{\text{G}}}^{2}$ = 0.0004) were significant. See Fig. 3. Including the group factors (order of conditions, natural versus reversed action–effect mapping) into the ANOVA design did not reveal any additional significant (main or interaction) effects. We followed up the non-significant interaction of prediction type and distractor presence by comparing the distractor interference RT costs (RT distractor present minus RT distractor absent) between the two prediction-type conditions. A Bayes factor analysis using Cauchy prior with a recommended scale of 0.707 yielded modest evidence for null effect: BF_01_ = 3.03 (Rouder et al., 2009). Finally, we conducted an ANOVA on the so-called inverse-efficiency scores, computed as median RT divided by accuracy (proportion of correct responses) as a potentially more sensitive, aggregate measure of performance (Townsend & Ashby, 1983). However, once again, this analysis yielded the same pattern of results, with a significant main effect of distractor presence (*F*[1, 26] = 63.8, *p* < 0.001), but a non-significant main effect of prediction-type and a non-significant interaction of these two factors (both *F* < 0.76, *p* > 0.39).

Thus, the follow-up experiment, which employed a potentially more sensitive, reaction time measure, likewise does not provide evidence in favor of a difference between the cue and action prediction conditions.

### Discussion

The present study was designed to examine two questions: (1) would the opportunity to predict the presence and location of an item that is task-irrelevant but attention-capturing by means of one’s own actions or by an informative cue interfere with task performance to a greater degree, as posited by the ‘attentional white bear’ hypothesis, or to a lesser degree, relative to when no prediction regarding the distracting item is possible? And (2) would the type of predictive information influence the degree to which the distractor interferes with task performance, specifically: is there evidence for a special role of motor stimulus identity prediction, as posited by optimal motor control theories, or is non-motor identity prediction sufficient for explaining the effect of distractor predictability on performance?

To examine these questions, we adapted an additional-singleton compound visual search task. In the first experiment, the search displays were presented only for a limited exposure duration and then immediately overwritten with post-display masks. Using this task design, we opted for a measure of distractor interference in terms of accuracy, rather than RT, costs, which is arguably better suited to capture effects arising at early, perceptual processing stages of attentional stimulus selection and discrimination, unaffected by later, post-selective processes of response selection and execution (Santee & Egeth, 1982). Of note, our study is one of only very few that successfully demonstrated attentional capture using this type of paradigm and measure. In the second experiment, display was presented until response and reaction times were measured, to examine whether the results obtained in Experiment 1 would be generalizable to another dependent variable, the reaction time measure. To address our research questions, we manipulated the way in which the presence and location of the distractor was predicted, namely, by providing participants with either an explicit informative external cue or making them internally generate a prediction in terms of the anticipated effect of a motor action they chose to perform. In all conditions, we controlled for factors such as the presence of an action, cognitive load, temporal predictability, and temporal control, which are common confounds in other studies on action–effect prediction (Hughes et al., 2013), to isolate the specific effects of non-motor and motor stimulus identity prediction. In this respect, we believe our study to be unique in the literature on the potential influences of motor prediction on attention.

In the first experiment, we found that for both prediction by an action and prediction by a cue, the distractor interference was reduced, compared to a (non-predictive) baseline condition. Because our action and external cues provided predictive information simultaneously about the presence and location of the distractor, future studies are needed to disentangle the respective contribution of these two aspects of prediction. Of note, the interference reduction was of a comparable magnitude whether the distractor was predicted by an external cue or by the choice of an action. Predictive information of either type about the absence of a distractor had no noticeable effect compared to the baseline, suggesting that the prediction indeed influenced the processing of the distractor item, rather than the performance improvement being due to some other facilitatory processes related to the provision of predictive information as such.

**Fig. 4:**
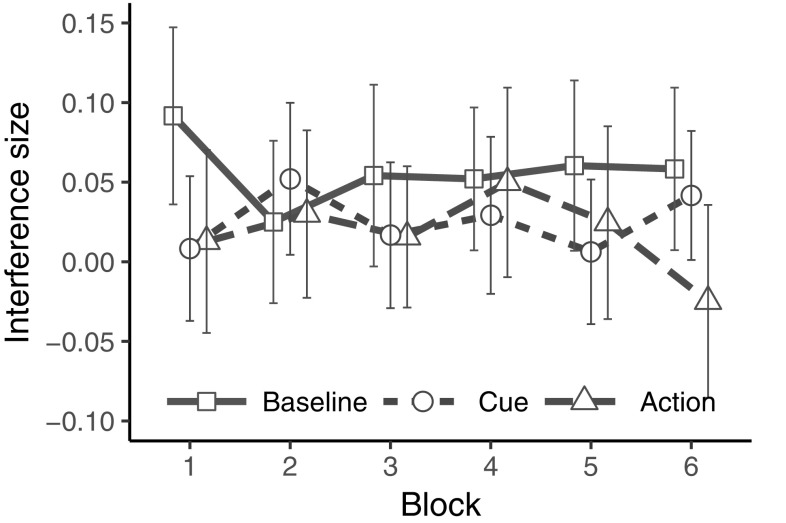
Distractor interference as a function of block (time) for each prediction-type condition. Interference magnitude is calculated as mean accuracy for distractor-absent minus distractor-present trials. There were six blocks (of 32 trials each) in each condition. Error bars depict 95% confidence intervals for the mean corrected for dependence in measurements (Morey, 2008)

Our attempt to capture, as well as possible, any specific effects of motor stimulus identity (action–effect) prediction came with a methodological cost, namely, presentation of the three prediction conditions in a fixed order. In particular, the action prediction condition had to be administered last because of the action–effect association (learning) phase that was required for this condition. Implementing this phase earlier on in our within-participants design would have influenced any other (i.e., the baseline and/or cue prediction) condition(s) participants would have performed after it (e.g., pressing the left button in a baseline condition performed after the action condition might have attenuated the intensity of a stimulus that happened to occur at the location previously associated with this action). However, our results provide no evidence that there was a learning effect within the three conditions, that is, there was no systematic reduction of distractor interference with increasing time on a particular task (prediction) condition (see the Appendix and Fig. 4)—possibly owing to the long exposure to distractors in the ‘exposure’ (or ‘association’) phase before each condition proper and the number of practice (96) and staircase (on average 96) trials at the start of the experiment (cf. Müller et al., 2009). In addition, across conditions, it is unlikely the change in task between the baseline and the cue and action prediction conditions as such brought about a step-like change in performance, due to some factor other than the predictive information provided by the cues, such as novelty or increased arousal. First of all, there was no difference in performance on distractor-absent trials among the three conditions, and for distractor-present trials, any increase in general arousal would, arguably, have led to increased distractor interference (assuming that arousal would have boosted the saliency of the distractor as well as that of the target; e.g. Zou, Muller, & Shi, 2012), rather than the reduction in interference we actually observed.

In any case, we do not believe that our main conclusions with regard to the two questions we set out to answer were compromised by our sequential condition order. First, our results clearly show that distractor prediction did not cause an ‘attentional white bear’ (AWB) effect: the AWB hypothesis predicts a performance cost associated with the cues (i.e., reduced accuracy on distractor-present trials in the cue- and action prediction conditions relative to the baseline), rather than the performance benefit that we actually observed. Second, optimal motor control theories predict that action–effect prediction has a specific, namely, an attenuating effect on the predicted stimulus, over and above the effect of cue prediction. However, we failed to find a significantly greater interference reduction for the action prediction versus the cue prediction condition—which may be taken to argue against optimal motor control theories (as further discussed below). However, despite having evidence favoring the null hypothesis (BF_01_ = 4.22), there was a small numerical difference and we cannot definitely rule out that self-generated action cues may be somewhat more effective in reducing interference than external cues (a difference we may have been unable to detect with the presented experimental designs and sample sizes).

#### Cue prediction and attentional white bear

With respect to prediction by external cue, participants were told they could use the cue information in any way that could help them perform the task better. Although most people reported no consistent usage of the information provided by the cue (see Appendix), the cue clearly had a positive effect on performance for most participants. This indicates that the external cue was actually being used by the majority of participants, without them being explicitly aware of this, perhaps in automatic manner, even without some kind of association phase as implemented in the action prediction condition. This is consistent with previous reports that people can extract cue information without being aware of this (Decaix, Siéroff, & Bartolomeo, 2002; Peterson & Gibson, 2011). A similar case can be made for the action prediction condition, in which participants presumably lacked a reason to deliberately and consciously guide their attention according to the button they pressed (although participants were not explicitly questioned about this at the end of the experiment).

#### Action–effect prediction processes

The difference in performance between the baseline and cue prediction conditions was supposed to reveal the influence of what Hughes et al. (2013) referred to as ‘non-motor identity prediction’ processes, that is, predicting the stimulus (and its properties) in a general manner (not necessarily related to motor processes). And importantly, any difference between the cue- and action prediction conditions was supposed to directly reflect the contribution of specific ‘motor identity prediction’ processes, in line with optimal motor control-based theories (Waszak et al., 2012; Wolpert & Flanagan, 2001). We failed to observe such an additional effect; rather, both types of prediction resulted in very similar effects, both in terms of the overall interference reduction as well as spatial distance effects (see Appendix). While we cannot definitely rule out that this null difference is simply a false negative finding (owing to lack of statistical power), we did achieve a power of 0.82 and 0.78 in our two experiments for observing an effect of the expected size and our Bayes factor analyses revealed more evidence for the null hypothesis of no effect versus the hypothesis of an effect.

Conceivably, our design may have been too different from that of Cardoso-Leite et al. (2010) in that instead of providing predictive information about a near-threshold stimulus our distractor was a highly salient display item. Forward model theories postulate that predicted sensory consequences of self-generated actions are subject to sensory attenuation, but the specific mechanism bringing about this attenuation is unclear. It is possible that such sensory signals are attenuated in a non-linear fashion, depending on the original strength of the stimulus, such that, for instance, very salient stimuli cannot be attenuated. However, Reznik, Henkin, Levy, and Mukamel (2015) found that while self-produced supra-threshold auditory stimuli were attenuated, near-threshold stimuli were enhanced. If their finding generalizes to the visual domain, our salient distractor should be subject to sensory attenuation. Another nonlinearity, described by Zehetleitner et al. (2013), may also make it possible that the sensory strength of the distractor was actually attenuated by motor prediction, but not enough to measurably reduce attentional capture (over and above the reduction with external cues). Zehetleitner et al. (2013) showed that the probability with which a distractor captures attention on a given trial is a psychometric function of the difference in salience between the distractor and the target: if the distractor is much more salient than the target, a small decrease in distractor salience—for instance due to the presumed attenuation of the sensory consequences of self-generated actions—would not translate into any, or only a very small, reduction of the probability of attentional capture.

Overall, while we cannot exclude existence of a sensory attenuation effect for action-specific, motor-identity prediction (Hughes et al., 2013), we observe no evidence in its favor in our experiments. We may only speculate that a more general mechanism may be engaged in both the action and cue prediction conditions. A highly prominent proposal of such a general principle is ‘predictive coding’, or, more generally, ‘predictive processing’ (Clark, 2013) and we therefore believe it is worth discussing how our results may fit into it.

On this view, only prediction errors are propagated to higher levels in a processing hierarchy, and this signal should thus be lower for a predicted than an unpredicted distractor, which could cause sensory attenuation. Importantly, the prediction errors are also weighted by their expected precision, where this precision weighting is generally taken as corresponding to the cognitive-psychological concept of attention (Feldman & Friston, 2010; Hohwy, 2012). Exactly what expected precision should be assigned to a salient but task-irrelevant distractor remains an open issue. Multiple factors come into play here. It has been proposed that task-irrelevant stimuli have reduced expected precision (Kok, Rahnev, Jehee, Lau, & de Lange, 2012). By contrast, we are thought to have a prior expectation (innate or acquired) that strong stimuli have a high signal-to-noise ratio and are thus more precise (Feldman & Friston, 2010). Arguably, therefore, the theory cannot readily answer the critical question whether prediction of the distractor would make it more or less interfering. What the theory would predict is that both cue- and action prediction should influence processing in a very similar manner, because both sources of prediction have the same accuracy, namely 100%, and also no variability in prediction errors—that is, they have the same precision. However, the theory also allows for a potential additional effect of action-specific prediction: The principle of ‘active inference’ posits that we need to decrease the precision of proprioceptive and somatosensory states to make a movement possible (Brown et al., 2013), though it remains unclear whether, how, and to what extent this might also concern visual processing.

Note though that our results are merely consistent with ‘predictive processing’, and it could be objected that this framework can accommodate all manners of possible result patterns. Despite the promises of this framework, we see it as still young and not yet sufficiently developed—especially with regard to explaining attentional phenomena (Ransom, Fazelpour, & Mole, 2017). Better, and ideally computationally explicit, models are thus required to derive more concrete testable predictions. For instance, Kok, Rahnev, et al. (2012) proposed a model of how attention interacts with prediction in a Posner-type cueing scenario—though their model essentially equates attention with task relevance, as they consider only prediction of task-relevant information. Our data on the interaction of attention and prediction of task-irrelevant stimuli might thus be useful for testing future, more complete models.

## Conclusions

In sum, the present study contributes another piece to the growing picture of how prediction by our own actions or by environmental (i.e., external) cues can improve attentional selection, even in the case of salient, but task-irrelevant distracting stimuli. Our findings show that both external cues and internally generated predictions similarly attenuate the negative impact of distracting stimuli on the efficiency of attentional selection. However, the data do not support the idea of a “special status” of motor-specific predictions playing a role in our experiments. Overall, the pattern observed can be explained within the context of the predictive coding framework, although it does not exclude other theoretical accounts. This illustrates that attributing effects such as sensory attenuation to specific prediction processes (such as motor prediction) is methodologically challenging, which should be kept in mind when designing experiments on these topics and interpreting their results.

## Figures and Tables

**Fig. 5:**
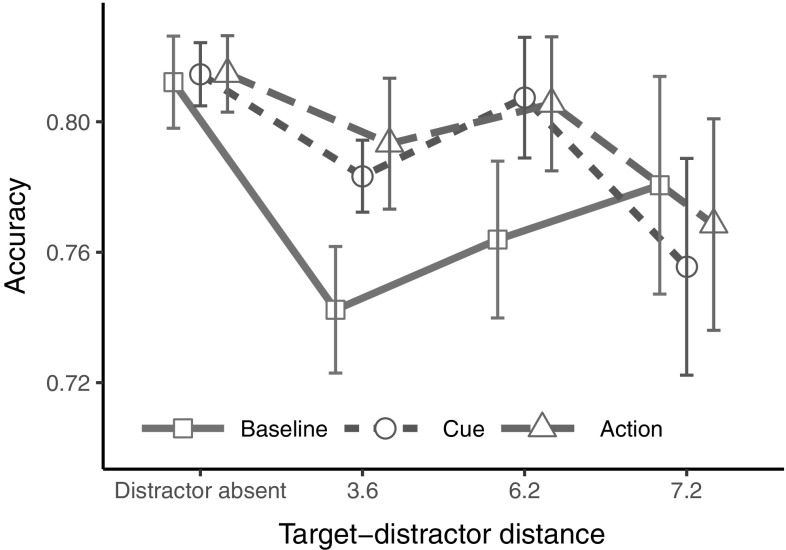
Effects of target-to-distractor distance on performance accuracy. Distances, in degrees of visual angle, apply only to distractor-present trials. Error bars depict 95% confidence intervals for the difference between means corrected for dependence in measurements (Morey, 2008). Error bar size is influenced by the differing numbers of trials for the various levels of distance (on average, per participant, 95, 44, 37, and 15 trials for distractor-absent, and 3.6°, 6.2°, and 7.2° distances on distractor-present trials, respectively)

## Data Availability

The data and analysis scripts used to produce the reported results are publicly available from the OSF repository: 10.17605/OSF.IO/ZBC78.

## Appendix: Additional Analyses of Experiment 1

### Learning effects

Because we presented conditions in a fixed sequence, it is possible that the improvement in accuracy in the presence (vs. the absence) of a distractor could be explained by simple learning. This would manifest itself as an effect of time on the magnitude of distractor interference in at least one of the conditions; specifically, interference would decrease as a function of time. To examine for such an effect, we conducted an ANOVA on interference magnitude (accuracy on distractor-absent minus distractor-present trials) with the within-participants factor prediction-type condition. Time was represented by a numeric within-participants variable “block number” (there were 6 blocks of trials in each condition). While the main effect of prediction-type condition was (still) significant (*F*[2, 58] = 4.52, *p* = 0.015, $\eta _{{\text{G}}}^{2}$ = 0.0538), the main effect of block number was not (*F*[1, 29] = 0.289, *p* = 0.595, $\eta _{{\text{G}}}^{2}$ = 0.0019), that is, the overall slope of interference size per block was not significantly different from zero; in addition, there was no interaction of block number with prediction-type condition (*F*[2, 58] = 0.235, *p* = 0.791, $\eta _{{\text{G}}}^{2}$ = 0.0036), that is, the slopes do not significantly differ between any of the three conditions. See Fig. 4. Although this does not rule out that there was some such gradual learning effect, it alone could not explain the observed reduction in distractor interference in the cue and action prediction conditions. In addition, we cannot exclude the presence of step-wise learning or other similar effects between the conditions.

### Sensorimotor contingencies

It is possible that the reduction in distractor interference in the action prediction condition, rather than being due to the learned sensorimotor associations, was actually driven by either that half of the participants for whom the mapping between actions and effects was “natural” (left key caused distractor at top-left location) or the other half with the “inverse” mapping (left key caused distractor at bottom-right location). To test for this possibility, we performed a mixed-design two-way ANOVA (on the action prediction condition data) with accuracy as the dependent measure, distractor presence as a within-participants factor, and contingency group (natural versus inverse) as between-participants factor. Neither the main effect of the contingency group (*F*[1,28] = 1.407, *p* = 0.246, $\eta _{{\text{G}}}^{2}$ = 0.043, $\eta _{{\text{p}}}^{2}$ = 0.048) nor, importantly, the interaction of contingency group with distractor presence (*F*[1,28] = 0.593, *p* = 0.448, $\eta _{{\text{G}}}^{2}$ = 0.0022, $\eta _{{\text{p}}}^{2}$ = 0.021) were significant, although distractor interference was numerically lower in the inverse group (*M* = 0.011, SD = 0.047) than in the natural group (*M* = 0.026, SD = 0.060). Therefore, the observed effects in the action prediction condition, rather than being sufficiently explained by the nature of the action–effect coupling, are more likely attributable to the learned sensorimotor contingencies.

With regard to theories proposing an inherent relation between action and attentional orienting (e.g., the ‘premotor theory of attention’; Rizzolatti, Riggio, & Sheliga, 1994), it is possible that it mattered whether the laterality of the (key press) action and the distractor location were congruent or incongruent. To examine this, we selected distractor-present trials that were started by a left- or a right-sided action (i.e., we disregarded trials started with a neutral action) and analyzed them in terms of the factor ‘action-distractor lateral congruence’ (‘ipsilateral’ when the side of the distractor was congruent with that of the action, and ‘contralateral’ when not). Note that for the action prediction condition, this factor maps directly onto the factor ‘contingency group’ analyzed above (ipsilateral for the natural and contralateral for the inverse mapping)—so that it is unsurprising that, again, we found no significant difference (F[1, 28] = 2.18, *p* = 0.151, $\eta _{{\text{G}}}^{2}$ = 0.072) between the two groups (ipsilateral distractors, *M* = 0.776, SD = 0.081; contralateral distractors, *M* = 0.817, SD = 0.074). For the baseline and cue prediction conditions, although there was an overall numerical difference between ipsilateral (*M* = 0.781, SD = 0.099) and contralateral trials (*M* = 0.759, SD = 0.080), the main effect did not reach significance (*F*[1, 29] = 1.675, *p* = 0.206, $\eta _{{\text{G}}}^{2}$ = 0.012) and there was no interaction with condition (*F*[1, 29] = 0.156, *p* = 0.696, $\eta _{{\text{G}}}^{2}$ < 0.001). We conducted an analogous analysis for the factor “action-target lateral congruence”. Given that in the action prediction condition, pressing the left or the right key always led to a distractor-present trial, we first analyzed only distractor-present trials (in all three conditions) per the factors of lateral congruence and prediction type. Second, we analyzed both distractor-present and -absent trials per action-target lateral congruence and prediction-type condition, but only in the baseline and cue prediction conditions. None of the main effects and interactions involving ‘lateral congruence’ were significant (all *F* < 0.54, *p* > 0.58). In sum, we found no evidence that the laterality of the actions per se influenced attentional selection and performance in our paradigm.

### Cue prediction condition

It is important to rule out that in the cue prediction condition, participants, after seeing a directional cue (left, right, or neutral), tended to choose the congruent key to start the trial (e.g., press left key when seeing a left cue), so that the action would be predictive of the distractor to some degree in this condition as well. This did not turn out to be the case: there was no association between the cue and the keys participants pressed (*Χ*^2^[4] = 1.388, *p* = 0.85), consistently with the instructions, see Table 3.

**Table 3:** Relative frequencies of actions starting the trial per cue type in the cue prediction condition of Experiment 1. Numbers in columns are means of individual percentages of actions that were produced when seeing a given cue type

Cue	Key pressed		
	Neutral	Left	Right
Neutral	48.6	25.7	25.7
Left	48.3	24.8	26.9
Right	49.6	24.7	25.8

We used a questionnaire to ask participants how they think they utilized or were influenced by the information provided by the cue. Only three participants reported a consistent influence of the cue (one reported feeling consistently captured, mean interference size in the cue prediction condition = 0.063; two reported that they had tried to ‘look away’, 0.083 and − 0.042, respectively); 17 participants said they had completely ignored the cue (*M* = 0.024) and the remaining 10 participants reported that they had occasionally looked in the direction of the cued location or away from it, started to ignore it after a few trials, or were not sure how they had used the cue (*M* = 0.026). Overall, there seems to be no clear correspondence between the reports and actual interference size.

### Distance effects

A variety of studies have reported distance effects in our type of paradigm, such as an inhibitory surround around a focus of attention (Gaspar & McDonald, 2014; Hopf et al., 2006; Tombu & Tsotsos, 2008). Hence, as an exploratory analysis, we split distractor-present trials according to the distance between the target and distractor. Visual inspection revealed the distance effects to be closely similar in the cue and action prediction conditions, as compared to the baseline condition (Fig. 5). However, an ANOVA on mean accuracies with the within-participants factors prediction-type condition and distance (as a categorical variable) did not reveal any significant effects (main effects: both *p* > 0.15; interaction: *F*[4, 116] = 1.99, *p* = 0.10). This may be owing to noisy estimates of accuracy at the longest distance (7.2°), due to a low number of trials for this distance. Visual inspection also suggested lower accuracy for targets closest (3.6°) to distractor compared to medium distances (6.2°), consistent with the inhibitory surround effect. Accordingly, we limited further analysis to these two distances (of 3.6° and 6.2°). The corresponding ANOVA revealed the main effect of prediction-type condition to be significant (*F*[2, 58] = 8.75, *p* < 0.001), whereas the main effect of distance only approached significance (*F*[1, 29] = 3.53, *p* = 0.07) and the interaction was non-significant (*F*[2, 58] = 0.141, *p* = 0.87). Follow-up tests comparing pairs of conditions revealed the main effect of prediction type to be present when (separately) comparing the cue prediction and the action prediction conditions versus the baseline (*F*[1, 29] = 13.5, *p* < 0.001, and, respectively, *F*[1, 29] = 11.23, *p* = 0.002), but not when comparing the cue versus the action prediction conditions (*F*[1, 29] = 0.13, *p* = 0.72). We can therefore conclude that prediction improved processing of targets at close-to-medium distances from the distractor in a similar way for both types of prediction.^2^

## Footnotes

1 Cardoso-Leite et al. (2010) reported a significant sensitivity reduction when the stimuli were predicted by an action (d_z_ = 0.793) but not when they were predicted by a tone (d_z_ = 0.247). Although both effects were investigated in two different groups of participants (of unequal size), we estimated the additional effect of action prediction on top of cue prediction as the difference between these two effects (d_z_ = 0.546).

## References

[CR1] Arita JT, Carlisle NB, Woodman GF (2012). Templates for rejection: Configuring attention to ignore task-irrelevant features. Journal of Experimental Psychology. Human Perception and Performance.

[CR2] Bacon, W. F., & Egeth, H. E. (1994). Overriding stimulus-driven attentional capture. *Perception* & *Psychophysics, 55*(5), 485–496. http://www.ncbi.nlm.nih.gov/pubmed/8008550. 10.3758/bf03205306 8008550

[CR3] Baess P, Horváth J, Jacobsen T, Schröger E (2011). Selective suppression of self-initiated sounds in an auditory stream: An ERP study. Psychophysiology.

[CR4] Beck V, Luck S, Hollingworth A (2011). The implementation of an exclusionary attentional template: Direct Versus indirect cueing. Journal of Vision.

[CR5] Blakemore SJ, Frith CD, Wolpert DM (1999). Spatio-temporal prediction modulates the perception of self-produced stimuli. Journal of Cognitive Neuroscience.

[CR6] Brown H, Adams R, Parees I, Edwards M, Friston K (2013). Active inference, sensory attenuation and illusions. Cognitive Processing.

[CR7] Buckolz E, Guy S, Khan M, Lawrence G (2006). Can the location negative priming process operate in a proactive manner?. Psychological Research Psychologische Forschung.

[CR8] Cardoso-Leite P, Mamassian P, Schütz-Bosbach S, Waszak F (2010). A new look at sensory attenuation. Action–effect anticipation affects sensitivity, not response bias. Psychological Science.

[CR9] Chao H-F (2010). Top-down attentional control for distractor locations: the benefit of precuing distractor locations on target localization and discrimination. Journal of Experimental Psychology. Human Perception and Performance.

[CR10] Christ SE, Abrams RA (2006). Abrupt onsets cannot be ignored. Psychonomic Bulletin & Review.

[CR11] Clark A (2013). Whatever next? Predictive brains, situated agents, and the future of cognitive science. The Behavioral and Brain Sciences.

[CR12] Decaix C, Siéroff E, Bartolomeo P (2002). How voluntary is ‘voluntary’ orienting of attention?. Cortex.

[CR13] Dhawan S, Deubel H, Jonikaitis D (2013). Inhibition of saccades elicits attentional suppression. Journal of Vision.

[CR14] Duncan J, Posner MI, Marin O (1985). Visual search and visual attention. Attention and performance XI.

[CR15] Eimer M, Kiss M (2008). Involuntary attentional capture is determined by task set: evidence from event-related brain potentials. Journal of Cognitive Neuroscience.

[CR16] Feldman H, Friston KJ (2010). Attention, uncertainty, and free-energy. Frontiers in Human Neuroscience.

[CR17] Friston K (2011). What is optimal about motor control?. Neuron.

[CR18] Gallagher S (2000). Philosophical conceptions of the self: implications for cognitive science. Trends in Cognitive Sciences.

[CR19] Gaspar JM, McDonald JJ (2014). Suppression of Salient Objects Prevents Distraction in Visual Search. Journal of Neuroscience.

[CR20] Gibson BS, Jiang Y (1998). Surprise! An unexpected color singleton does not capture attention in visual search. Psychological Science.

[CR21] Herwig A, Waszak F (2012). Action–effect bindings and ideomotor learning in intention- and stimulus-based actions. Frontiers in Psychology.

[CR22] Hickey C, McDonald JJ, Theeuwes J (2006). Electrophysiological evidence of the capture of visual attention. Journal of Cognitive Neuroscience.

[CR23] Hohwy J (2012). Attention and conscious perception in the hypothesis testing brain. Frontiers in Psychology.

[CR24] Hommel, B., Pratt, J., Colzato, L., & Godijn, R. (2001). Symbolic control of visual attention. *Psychological Science, 12*(5), 360–365. http://pss.sagepub.com/content/12/5/360.short. 10.1111/1467-9280.00367 11554667

[CR25] Hopf J-M, Boehler CN, Luck SJ, Tsotsos JK, Heinze H-J, Schoenfeld MA (2006). Direct neurophysiological evidence for spatial suppression surrounding the focus of attention in vision. Proceedings of the National Academy of Sciences of the United States of America.

[CR26] Huffman G, Rajsic J, Pratt J (2017). Ironic capture: top-down expectations exacerbate distraction in visual search. Psychological Research Psychologische Forschung.

[CR27] Hughes G, Desantis A, Waszak F (2013). Mechanisms of intentional binding and sensory attenuation: The role of temporal prediction, temporal control, identity prediction, and motor prediction. Psychological Bulletin.

[CR28] Kiss M, Grubert A, Petersen A, Eimer M (2012). Attentional capture by salient distractors during visual search is determined by temporal task demands. Journal of Cognitive Neuroscience.

[CR29] Kok P, Rahnev D, Jehee JFM, Lau HC, de Lange FP (2012). Attention reverses the effect of prediction in silencing sensory signals. Cerebral Cortex.

[CR30] Lahav A, Makovski T, Tsal Y (2012). White bear everywhere: exploring the boundaries of the attentional white bear phenomenon. Attention, Perception & Psychophysics.

[CR31] Lamy D, Egeth HE (2003). Attentional capture in singleton-detection and feature-search modes. Journal of Experimental Psychology. Human Perception and Performance.

[CR32] Morey RD (2008). Confidence intervals from normalized data: A correction to cousineau (2005). Tutorials in Quantitative Methods for Psychology.

[CR33] Müller HJ, Geyer T, Zehetleitner M, Krummenacher J (2009). Attentional capture by salient color singleton distractors is modulated by top-down dimensional set. Journal of Experimental Psychology. Human Perception and Performance.

[CR34] Müller HJ, Reimann B, Krummenacher J (2003). Visual search for singleton feature targets across dimensions: Stimulus- and expectancy-driven effects in dimensional weighting. Journal of Experimental Psychology. Human Perception and Performance.

[CR35] Munneke J, Van der Stigchel S, Theeuwes J (2008). Cueing the location of a distractor: an inhibitory mechanism of spatial attention?. Acta Psychologica.

[CR36] Olivers CNL (2009). What drives memory-driven attentional capture? The effects of memory type, display type, and search type. Journal of Experimental Psychology. Human Perception and Performance.

[CR37] Peterson SA, Gibson TN (2011). Implicit attentional orienting in a target detection task with central cues. Consciousness and Cognition.

[CR38] Pickering MJ, Clark A (2014). Getting ahead: forward models and their place in cognitive architecture. Trends in Cognitive Sciences.

[CR39] Rangelov D, Müller HJ, Zehetleitner M (2013). Visual search for feature singletons: Multiple mechanisms produce sequence effects in visual search. Journal of Vision.

[CR40] Rangelov D, Müller HJ, Zehetleitner M (2017). Failure to pop out: Feature singletons do not capture attention under low signal-to-noise ratio conditions. Journal of Experimental Psychology: General.

[CR41] Ransom M, Fazelpour S, Mole C (2017). Attention in the predictive mind. Consciousness and Cognition.

[CR42] Reznik D, Henkin Y, Levy O, Mukamel R (2015). Perceived loudness of self-generated sounds is differentially modified by expected sound intensity. PLoS One.

[CR43] Richters DP, Eskew RT (2009). Quantifying the effect of natural and arbitrary sensorimotor contingencies on chromatic judgments. Journal of Vision.

[CR44] Rizzolatti G, Riggio L, Sheliga BM, Umiltá C, Moscovitch M (1994). Space and selective attention. Attention and performance XV.

[CR45] Rouder JN, Speckman PL, Sun D, Morey RD, Iverson G (2009). Bayesian t tests for accepting and rejecting the null hypothesis. Psychonomic Bulletin & Review.

[CR46] Ruff CC, Driver J (2006). Attentional preparation for a lateralized visual distractor: Behavioral and fMRI evidence. Journal of Cognitive Neuroscience.

[CR47] Santee, J. L., & Egeth, H. E. (1982). Do reaction time and accuracy measure the same aspects of letter recognition? *Journal of Experimental Psychology. Human Perception and Performance, 8*(4), 489–501. http://www.ncbi.nlm.nih.gov/pubmed/6214603. 10.1037//0096-1523.8.4.489 6214603

[CR48] Sauter, M., Liesefeld, H. R., Zehetleitner, M., & Müller, H. J. (2018). Region-based shielding of visual search from salient distractors: Target detection is impaired with same- but not different-dimension distractors. Attention, Perception, & Psychophysics, 1–21. 10.3758/s13414-017-1477-4. 10.3758/s13414-017-1477-4 29299850

[CR49] Theeuwes J (1992). Perceptual selectivity for color and form. Perception & Psychophysics.

[CR50] Theeuwes J (2010). Top-down and bottom-up control of visual selection. Acta Psychologica.

[CR51] Theeuwes J, Atchley P, Kramer AF, Monsell S, Driver J (2000). On the time course of top-down and bottom-up control of visual attention. Control of cognitive processes: Attention and performance XVIII.

[CR52] Tombu M, Tsotsos JK (2008). Attending to orientation results in an inhibitory surround in orientation space. Perception & Psychophysics.

[CR53] Townsend JT, Ashby FG (1983). Stochastic modeling of elementary psychological processes.

[CR54] Tsal Y, Makovski T (2006). The attentional white bear phenomenon: the mandatory allocation of attention to expected distractor locations. Journal of Experimental Psychology. Human Perception and Performance.

[CR55] Van Doorn G, Hohwy J, Symmons M (2014). Can you tickle yourself if you swap bodies with someone else?. Consciousness and Cognition.

[CR56] Waszak F, Cardoso-Leite P, Hughes G (2012). Action effect anticipation: Neurophysiological basis and functional consequences. Neuroscience and Biobehavioral Reviews.

[CR57] Weiss C, Herwig A, Schütz-Bosbach S (2011). The self in action effects: Selective attenuation of self-generated sounds. Cognition.

[CR58] Wolpert, D. M., & Flanagan, J. (2001). Motor prediction. *Current Biology, 11*(18), R729–R732. http://yadda.icm.edu.pl/yadda/element/bwmeta1.element.elsevier-688d420e-176b-31e7-a3a0-556ee0ceb5c0/c/main.pdf. 10.1016/s0960-9822(01)00432-8 11566114

[CR59] Woodman GF, Luck SJ (2007). Do the contents of visual working memory automatically influence attentional selection during visual search?. Journal of Experimental Psychology. Human Perception and Performance.

[CR60] Wykowska A, Schubö A (2010). On the temporal relation of top-down and bottom-up mechanisms during guidance of attention. Journal of Cognitive Neuroscience.

[CR61] Wykowska A, Schubö A (2011). Irrelevant singletons in visual search do not capture attention but can produce nonspatial filtering costs. Journal of Cognitive Neuroscience.

[CR62] Yantis, S. (1993). Stimulus-driven attentional capture. *Current Directions in Psychological Science, 2*(5), 156–161. http://www.jstor.org/stable/10.2307/20182231. 10.1111/j.1467-8721.2008.00554.x PMC2681259 19444327

[CR63] Zehetleitner M, Koch AI, Goschy H, Müller HJ (2013). Salience-based selection: Attentional capture by distractors less salient than the target. PloS One.

[CR64] Zou H, Muller HJ, Shi Z (2012). Non-spatial sounds regulate eye movements and enhance visual search. Journal of Vision.

